# Iatrogenic vertebral artery injury during central line placement: a case report

**DOI:** 10.1093/jscr/rjaf947

**Published:** 2025-11-25

**Authors:** Osama Alahmadi, Hadeel Bin Shuiel, Muteb Alotaibi, Omer Elshaer, Abdul Rehman Zia Zaidi, Samer Koussayer

**Affiliations:** Department of Surgery, Al Noor Specialist Hospital, Al Third Ring Road, Al Hijrah District, Makkah 21955, Saudi Arabia; College of Medicine, AlFaisal University, Takhassusi Street, Al-Maather District, Riyadh 11533, Saudi Arabia; College of Medicine, AlFaisal University, Takhassusi Street, Al-Maather District, Riyadh 11533, Saudi Arabia; College of Medicine, AlFaisal University, Takhassusi Street, Al-Maather District, Riyadh 11533, Saudi Arabia; College of Medicine, AlFaisal University, Takhassusi Street, Al-Maather District, Riyadh 11533, Saudi Arabia; Department of Surgery, King Faisal Specialist Hospital and Research Center, Al-Maather District, Riyadh 11533, Saudi Arabia

**Keywords:** central venous catheterization, vertebral artery injury, endovascular stent, interventional radiology, case report

## Abstract

Central venous catheterization is commonly performed in critically ill patients but carries risks, including arterial injury. Vertebral artery injury during central venous catheterization insertion is rare but can cause severe morbidity and mortality if not promptly recognized and managed. Early diagnosis and intervention are crucial to minimizing complications. A 55-year-old Arab male of Middle Eastern ethnicity with severe community-acquired pneumonia required intubation and vasopressor support. During an attempt to insert a central venous catheterization through the left internal jugular vein, the patient developed acute hypotension and a significant hemoglobin drop, indicating active bleeding. Computed tomography angiography confirmed a left vertebral artery injury. Endovascular repair with a covered balloon-expandable stent via brachial access led to immediate hemodynamic stabilization and bleeding control. This case highlights the importance of meticulous technique, monitoring, and advanced imaging. Vascular surgery consultation and imaging guide management. Endovascular procedures offer safer, less invasive options when performed by experienced interventionists.

## Introduction

Central venous catheterization (CVC) is a crucial and frequently performed procedure in critically ill patients for hemodynamic monitoring, medication administration, and fluid resuscitation [1, 2]. While generally safe, complications occur in ~1.5% of procedures, including infection, thrombosis, hemothorax, pneumothorax, and arterial injury [3, 4]. Arterial injuries during internal jugular vein CVC placement occur in 3%–9.5% of cases, with misplacement rates around 0.17% [5]. The carotid artery is most commonly affected, whereas vertebral artery injury (VAI) is rare, with an incidence below 1% [6].

Vertebral artery damage can result in pseudoaneurysm, dissection, arteriovenous fistula, or thromboembolic stroke [1]. The vertebral artery is anatomically divided into four segments (V1–V4), with V1 most susceptible to injury [7]. Clinical symptoms of VAI include neck pain, dizziness, headache, nausea, sensory deficits, or may be asymptomatic [8]. Computed tomography angiography (CTA) is the preferred non-invasive diagnostic tool due to high sensitivity and specificity [9]. Digital subtraction angiography remains the gold standard, but is more invasive and carries increased procedural risk [9].

Management strategies for VAI include catheter removal with compression, surgical repair, or endovascular intervention [10, 11]. The pull/pressure technique carries high risks of hematoma, stroke, and airway compromise, and is now considered suboptimal [12]. Surgical repair has historically been used, but is associated with higher morbidity and mortality [12]. Surgical intervention is reserved for expanding hematomas, failed endovascular attempts, or uncontrolled bleeding [12].

Recent advances have positioned endovascular therapy—such as stent grafting or embolization—as the preferred treatment due to high success rates and preservation of cerebral circulation [12].

We present a case of iatrogenic VAI during CVC placement successfully managed with endovascular stent grafting.

## Case presentation

A 55-year-old man with diabetes, hypertension, and mild systolic heart failure (LVEF 40%–45%) presented to the emergency department with 2 days of worsening shortness of breath, fever, productive cough, and pleuritic chest pain. Initial vitals revealed tachypnea, tachycardia, and hypoxia. Arterial blood gas showed acidosis (pH 7.25), hypercapnia (PaCO₂ 60 mmHg), and hypoxemia (PaO₂ 60 mmHg), despite 100% oxygen via a non-rebreather mask. He was intubated from respiratory failure.

He was transferred to the intensive care unit, where he became hypotensive and required vasopressor support. A central venous catheter was attempted via the left internal jugular vein. Soon after insertion, the patient became acutely hypotensive with blood pressure dropping to 60/40 mmHg, unresponsive to fluids. Hemoglobin dropped from 12 to 9.5 g/dl within 30 minutes, suggesting active hemorrhage.

**Figure 1:**
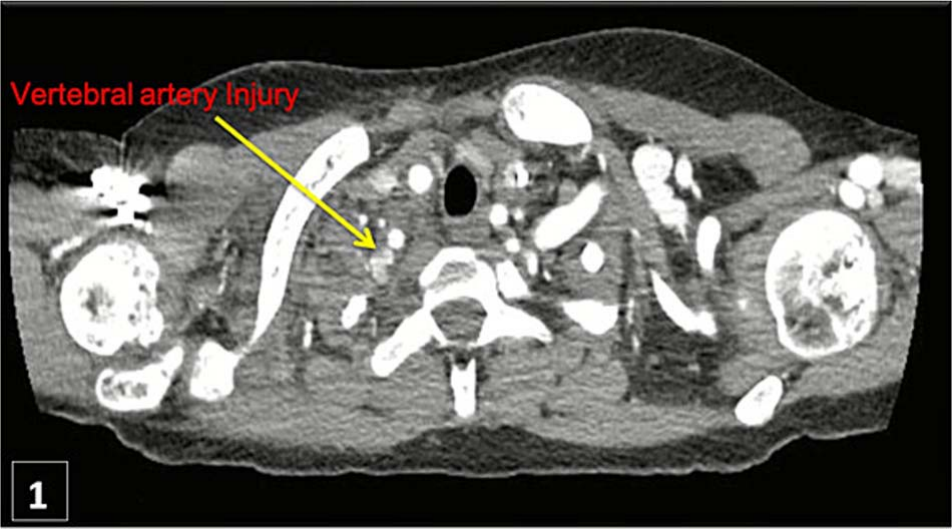
Thoracic CT angiogram shows contrast extravasation from left vertebral artery after CVC.

**Figure 2:**
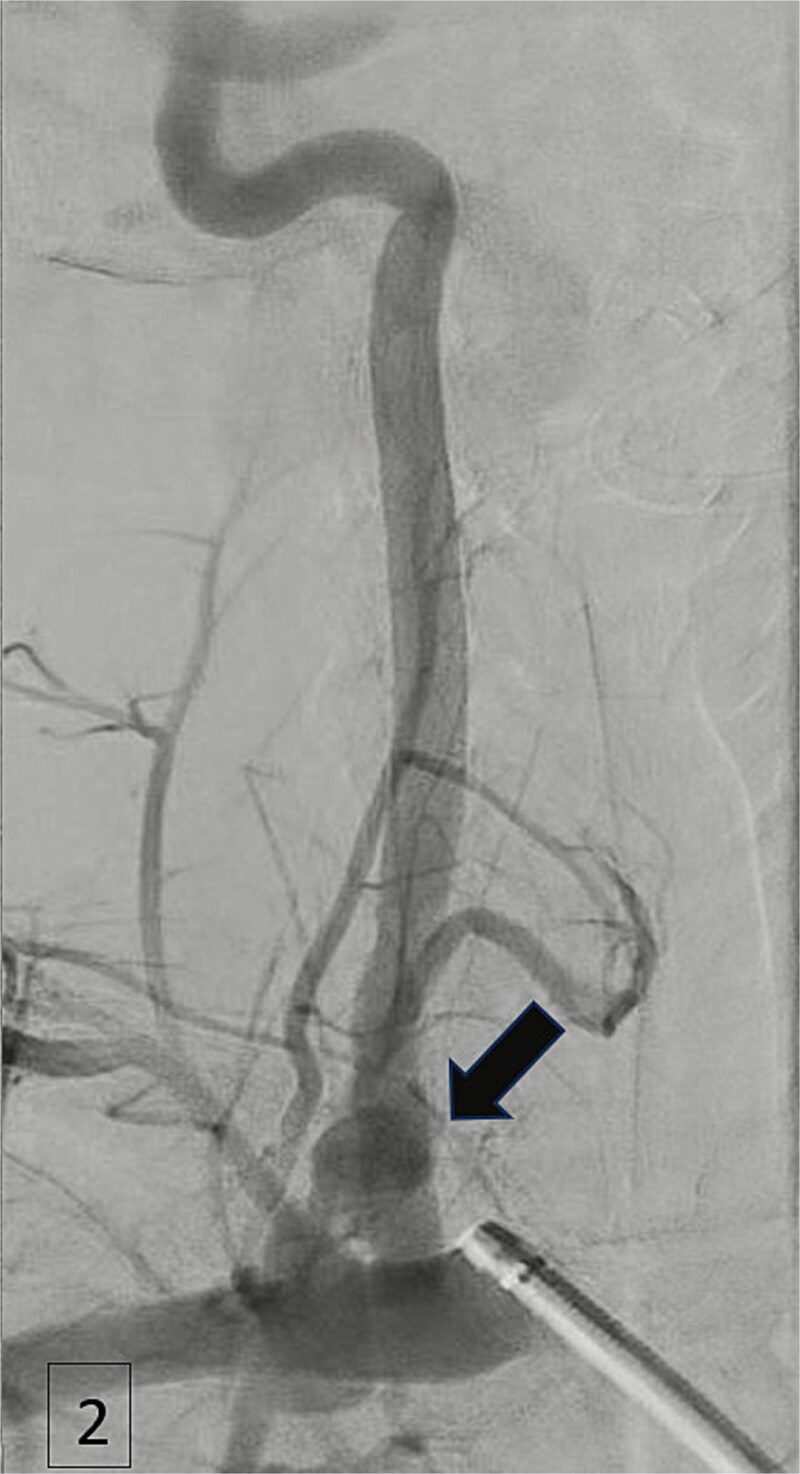
Angiogram demonstrating arterial injury following catheter insertion (arrow).

**Figure 3:**
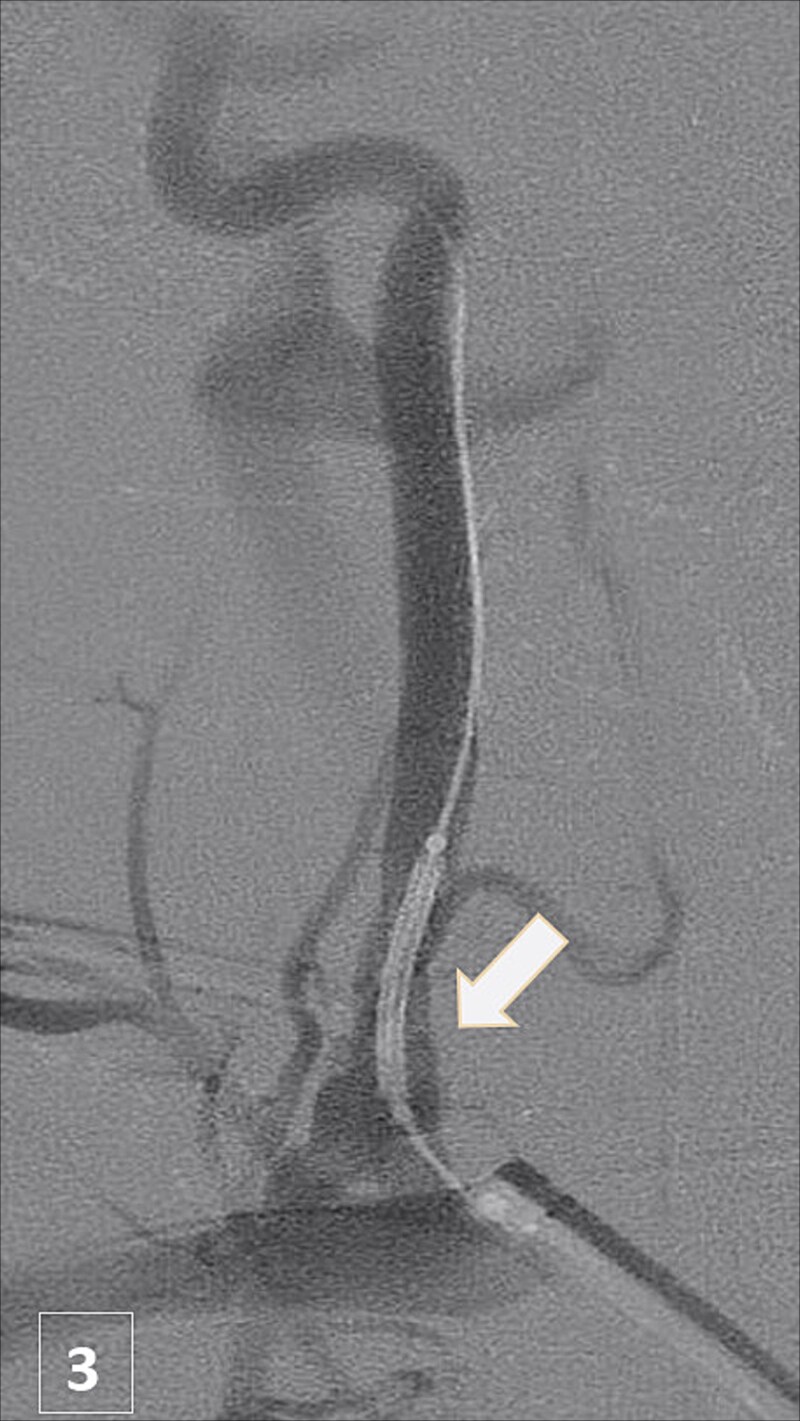
Post-stent angiogram showing complete sealing of injury with preserved flow.

**Figure 4:**
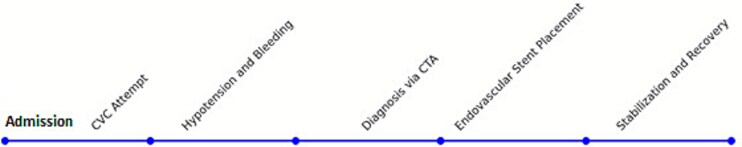
Timeline of clinical events for the management of iatrogenic VAI.

An urgent CTA revealed contrast extravasation from the proximal left vertebral artery, ~1 cm from its origin at the subclavian artery (Fig. 1). He was immediately taken to the angiography suite. Via left brachial access, a balloon-expandable covered stent (4 × 29 mm) was deployed, completely sealing the injury and restoring flow (Fig. 2). Post-procedure imaging confirmed no further extravasation (Fig. 3). The patient stabilized, vasopressors were weaned, and he was discharged without neurologic deficits. The patient’s clinical course and management are summarized in a timeline (Fig. 4).

## Discussion

VAI during CVC is exceedingly rare but can result in catastrophic complications if not recognized promptly [13]. Hemodynamic instability following catheter placement should raise concern for vascular injury. CTA offers rapid and accurate diagnosis with high sensitivity [13].

The pull-and-pressure technique—removing the catheter followed by manual compression—may be effective for compressible arteries, but is not ideal for deep vessels like the vertebral artery, and is associated with significant risks, including stroke and pseudoaneurysm [14]. In contrast, endovascular stent grafting is minimally invasive and has been shown to effectively control bleeding while preserving cerebral circulation [14].

Surgical repair is generally reserved for cases where endovascular treatment fails, or when there is damage to adjacent structures, expanding hematoma, or airway compromise [14]. However, surgery for VAI carries a mortality rate of up to 20% in unstable patients [14].

Our case supports existing evidence that endovascular stent grafting is a safe and effective treatment for iatrogenic VAI [15]. It underscores the need for a high index of suspicion, rapid imaging, and coordination between critical care, vascular surgery, and interventional radiology teams.

## Conclusion

Although VAI during central line placement is rare, it can result in life-threatening complications. Prompt recognition, vascular consultation, and advanced imaging are essential for optimal management. Endovascular treatment with covered stents offers a minimally invasive, effective solution, especially when performed by experienced operators.
